# Levodopa / opicapone as a complement to STN-DBS in clinical practice. A retrospective single-centre analysis.

**DOI:** 10.1016/j.ensci.2024.100530

**Published:** 2024-09-28

**Authors:** Moritz A. Loeffler, Philipp Klocke, Idil Cebi, Alireza Gharabaghi, Daniel Weiss

**Affiliations:** a Centre for Neurology, Department of Neurodegenerative Diseases, University of Tübingen, Hoppe-Seyler-Str. 3, 72076 Tübingen, Germany; b Hertie-Institute for Clinical Brain Research, University of Tübingen, Otfried-Müller-Straße 27, 72076 Tübingen, Germany; c Institute for Neuromodulation and Neurotechnology, University of Tübingen, Otfried-Müller-Straße 45, 72076 Tübingen, Germany

**Keywords:** Deep brain stimulation, Medication, COMT, Opicapone, Motor fluctuations, Denervation

## Abstract

**Objective:**

Deep brain stimulation of the subthalamic nucleus (STN-DBS) is a well-established treatment option in Parkinson's disease with motor and non-motor fluctuations allowing for postoperative reduction of dopaminergic medication. However, evidence is scarce on optimal medication adjustments following STN-DBS implantation. Opicapone allows for long-lasting inhibition of the catechol-*O*-methyltransferase (COMT) thereby enabling more constant dopaminergic stimulation compared to levodopa alone. However, especially COMT inhibitors are regularly discontinued after STN-DBS surgery. In this single-centre retrospective analysis, we aimed to analyse the clinical phenotype of patients selected for opicapone treatment following STN-DBS implantation and to define clinical determinants of patients requiring more intense dopamine-stabilising strategies after STN-DBS implantation.

**Methods:**

A patient cohort treated with STN-DBS + levodopa + opicapone (*n* = 16) was compared to an age-matched control cohort without opicapone treatment at baseline before and ≥ 5 months post-surgery. As main outcomes we assessed the MDS-UPDRS III and IV scores and reduction of the cumulative dopaminergic medication quantified by the levodopa equivalent dosages (LED).

**Results:**

Whilst the MDS-UPDRS III (median [min – max]) in patients with STN-DBS as well as anatomical electrode positions did not differ significantly between the opicapone 20 [4–40] and control cohort 14 [1–44], the patients selected for opicapone treatment showed a significantly higher degree of dyskinesias already preoperatively as reflected by a UPDRS-IV A subscore of 2 [0–4] compared to controls 0 [0–4]. Postoperatively, the opicapone cohort showed stronger motor fluctuations MDS-UPDRS IV 6 [0–14] compared to the controls 0 [0−10], albeit without statistical significance. Moreover, the opicapone cohort showed significantly less reduction of dopaminergic medication (−36.4 % vs. -46.2 % in the control cohort) following STN-DBS implantation independent from the intake of dopamine agonists.

**Conclusion:**

These results indicate a clinical phenotype characterised by more motor fluctuations requiring a more stable dopamine replacement therapy to address the patients' disease biology. In these cases, levodopa + COMT inhibition by opicapone represents a therapeutic approach but determination of the potential clinical benefit requires further prospective studies.

## Highlights

• Rare evidence on medication adjustments after STN-DBS implantation in Parkinson's Disease.
• COMT inhibitors are often discontinued after STN-DBS implantation.
• Patients with dyskinesias before DBS show less reduction in dopaminergic medication.
• This phenotype qualifies for opicapone as a complement to STN-DBS.
• Medications adjustments should be based on clinical determinants reflecting disease biology.

## 1 Introduction

Deep brain stimulation of the subthalamic nucleus (STN-DBS) is an established treatment option for Parkinson's disease (PD) improving quality of life in patients after the onset of dopaminergic motor and non-motor complications (1–4). Especially levodopa-sensitive symptoms are likely to exhibit a significant degree of relieve after STN-DBS implantation. Generally, STN-DBS allows for a significant reduction of the levodopa equivalent dose (LED) thereby lowering hyperdopaminergic side effects (5–7). However, dopaminergic replacement therapy is still required in combination to STN-DBS to achieve beneficial motor and non-motor outcome. Especially affective symptoms, axial symptoms and gait disturbances like freezing of gait (FOG) highly rely on constant dopamine serum levels (8, 9).

Conversely, clinical studies on how to adapt medication regimens in PD patients after STN-DBS are scarce. However, for beneficial clinical outcome it is crucial to identify clinical phenotypes that benefit from specific dopaminergic medication regimens as a supplement to STN-DBS. A first randomised single-blinded study has investigated the motor- and non-motor outcomes in patients with STN-DBS and either levodopa or dopamine agonist monotherapy. In the long-term follow-up 2.5 years after implantation, the majority of patients required a combination therapy of levodopa and dopamine agonists for stable symptom relief and control of adverse effects (10). The clinical role of other agents stabilising dopamine levels in combination with STN-DBS has not yet been analysed. The combination of levodopa and inhibitors of the catechol-*O*-methyltransferase (COMT) represents a clinically well-established approach to stabilise dopaminergic stimulation. However, earlier retrospective analyses suggested, that COMT inhibitors are preferentially discontinued after DBS surgery as part of the postoperative reduction of dopaminergic medication (11). Earlier on, COMT inhibition was primarily achieved using entacapone, which – upon oral delivery - has a short half-life limiting the stabilisation of dopamine plasma levels (12), and tolcapone which has potential side effects, particularly liver toxicity albeit in a minority of patients (13).

The third generation COMT inhibitor opicapone is characterised by a prolonged half-life compared to entacapone and is therefore considered to be particularly effective in stabilising levodopa plasma levels and reducing time in the OFF state of PD (12, 14–18). As a result, opicapone is considered a complement to STN-DBS. Although administered to patients in clinical practice, the combination of levodopa + opicapone has not been systematically investigated in the context of STN-DBS. In particular, it is not clearly defined which clinical phenotypes would benefit from a combination therapy with opicapone after STN-DBS implantation.

In this single-centre retrospective study we analysed the real-world use of opicapone in comparison to other medication regimens in PD patients with STN-DBS using longitudinally recorded clinical data. We aimed to identify patient characteristics requiring stabilisation of levodopa plasma levels with opicapone after STN-DBS.

## 2 Material and methods

### 2.1 Composition of the study cohort

We screened our institution's Parkinson's disease retrospective database (REDcap) for patients implanted with STN-DBS at the University Hospital Tübingen in the years 2014–2023 who were prescribed opicapone following authorisation by the European Medicines Agency in 2016. All patients were diagnosed with idiopathic Parkinson's disease according to the British Brain Bank criteria (19) and had received STN-DBS based on present indication criteria (1, 3). Postoperative intake of opicapone alongside clinical data of routine examinations before and ≥ 5 months post implantation were gathered. We focused on the examination of motor function and FOG, so that the minimum requirement for inclusion in the study cohort was the availability of a *Movement Disorders Society Unified Parkinson's Disease Rating Scale III (MDS-UPDRS III)* score at both examination timepoints. Further inclusion criteria were age 18–85 years and disease duration ≥3 years at the time of DBS implantation. Patients representing a severe pathological chronic or acute condition that might confound treatment effects or interpretation of the data were excluded from the analysis. All procedures conformed to the Declaration of Helsinki and were approved by our Institutional Review Board (040/2021BO2).

In all preoperative and most postoperative examinations *MDS-UPDRS I* (non-motor aspects of experiences of daily living), *MDS-UPDRS II* (motor aspects of experiences of daily living) and *MDS-UPDRS IV* (motor complications) were documented. Cognitive performance was scored using the *Montreal Cognitive Assessment (MoCA)*. Depressive symptoms were assessed by the *Beck Depression Inventory II (BDI-II)*. The overall disease stage was classified according to the *Modified Hoehn and Yahr Scale*. Additionally, we screened all patient histories for the presence of FOG and falls as a descriptive outcome and a correlate of advanced gait disorders. Dopaminergic medication was quantified by the LED before and after STN-DBS implantation (20, 21). A total of 16 patients (6 females, 10 males) met all inclusion criteria. The opicapone cohort had a mean age of 60.8 ± 11.3 years and mean disease duration of 11.3 ± 3.9 years (Table 1).

**Table 1:** Clinical characteristics of the patient cohorts. Baseline <6 months before DBS implantation.

ID	Sex	PD-Subtype	Age	Disease Duration	preOP	postOP
			*(years)*	*(years)*	Opicapone	Opicapone
Opi_1	m	AK	63	14	Yes	Yes
Opi_2	m	MX	59	12	no	yes
Opi_3	m	AK	53	7	no	yes
Opi_4	f	AK	54	10	no	yes
Opi_5	f	MX	75	9	no	yes
Opi_6	f	MX	60	9	no	yes
Opi_7	m	AK	53	5	no	yes
Opi_8	m	MX	61	12	no	yes
Opi_9	f	AK	59	17	yes	yes
Opi_10	m	AK	68	8	yes	yes
Opi_11	m	AK	62	11	yes	yes
Opi_12	m	MX	56	14	yes	yes
Opi_13	m	MX	62	8	yes	yes
Opi_14	f	MX	54	14	no	yes
Opi_15	m	MX	71	9	yes	yes
Opi_16	f	MX	62	21	yes	yes
Ctrl_1	m	MX	62	22	no	no
Ctrl_2	m	MX	61	23	no	no
Ctrl_3	m	MX	59	5	no	no
Ctrl_4	m	MX	52	7	no	no
Ctrl_5	m	MX	65	7	no	no
Ctrl_6	m	AK	65	15	no	no
Ctrl_7	m	MX	67	3	no	no
Ctrl_8	m	AK	61	16	no	no
Ctrl_9	m	AK	71	12	no	no
Ctrl_10	f	AK	61	14	no	no
Ctrl_11	f	MX	67	15	yes	no
Ctrl_12	m	MX	50	3	no	no
Ctrl_13	m	AK	59	10	no	no
Ctrl_14	f	MX	65	11	no	no
Ctrl_15	f	MX	61	10	no	no
Ctrl_16	f	MX	62	21	no	no
***Opi***			60.8 ± 11.3	11.3 ± 3.9	***Mean ± SD***	
***Ctrl***			61.8 ± 5.1	12.1 ± 6.2	***Mean ± SD***	

AR: Akinetic-rigid PD; MX: Mixed PD.

### 2.2 Composition of the control cohort

For the control cohort, we screened patients who underwent STN-DBS implantation between 2016 and 2023 but did not receive opicapone during the postoperative follow-up. To be eligible for inclusion in the control cohort, patients had to have documented clinical assessments both before and after DBS implantation, in particular at least the UPDRS III score. From this pool of eligible patients, we randomly selected (not in a fixed or sequential order) a sample that corresponded to the opicapone cohort (*n* = 16). We then checked the mean age of this sample at the time of DBS implantation (baseline analysis <6 months before DBS implantation). Our aim was to achieve the mean age of the opicapone cohort within a range of ±2 years. Therefore, the random selection process could be repeated until the mean age of the control cohort was within the targeted range. This ensured that the two groups had comparable age characteristics. (As the first random selection already fitted within the target age range, no repetition was necessary). The final control cohort consisted of 16 patients (5 females, 11 males) with a mean age of 61.8 ± 5.1 years and a mean disease duration of 12.1 ± 6.2 years at the time of STN-DBS implantation (Table 1).

### 2.3 Reconstruction of DBS electrode placement

Using both pre-operative MR images and post-operative CT images, the localisation of active contacts was assessed using an automated fusion algorithm iPlan 3.0 stereotaxy (BrainLab Inc., Munich, Germany) and manual marking of the active contacts. *X-*, *y-* and *z-*coordinates of each active contact of each electrode are given in relation to the midcommissural point in between the anterior and posterior commissure (Supplemental Table 1).

### 2.4 Statistical analyses

Both opicapone and control cohorts were examined at two defined timepoints: before and at ≥5 months post STN-DBS implantation. Clinical data was tested for normal distribution using Shapiro-Wilk-test and reported as mean mean ± standard deviation if parametric. Non-parametric data were reported as median [min – max]. Clinical data were compared within cohorts using Wilcoxon signed rank test and across cohorts with the Mann-Whitney *U* Test, respectively. Statistical significance was set at an alpha-level of 0.05. Data management was conducted using the REDcap platform (22) and Microsoft Excel 2019 (Microsoft, Redmond, WA, USA). Statistical analyses were performed using IBM SPSS statistics (IBM, Armonk, NY, USA). GraphPad PRISM 6.0 (GraphPad Software Inc., San Diego, CA, USA) was used for plotting the data.

## 3 Results

### 3.1 Dopaminergic medication and STN-DBS improved motor symptoms

**Fig. 1:**
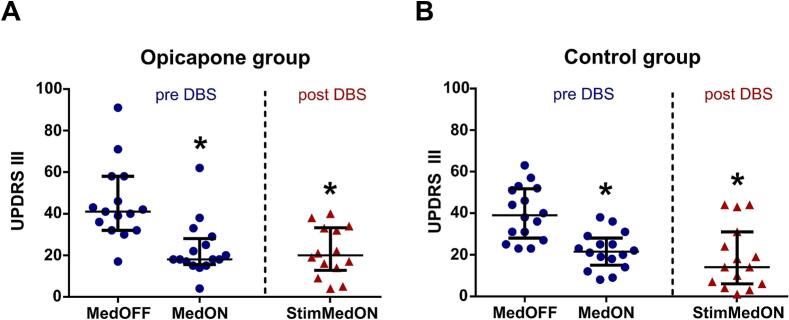
**Examination of motor function scored by MDS-UPDRS III. (A)** In the opicapone cohort the median MDS-UPDRS III in the MedOFF condition significantly decreased in MedON preoperatively and in the MedOFF-StimON condition postoperatively. **(B)** In the control cohort the median MDS-UPDRS III in the MedOFF condition significantly decreased in MedON preoperatively and in the MedOFF-StimON condition postoperatively. Data are displayed in scatter plots with the median and the interquartile range. Statistical test: sign test, p* < 0.05.

The overall motor outcome was assessed by scoring the MDS-UPDRS III. In the preoperative clinical evaluation of the opicapone cohort (Table 2), all patients showed sufficient levodopa response. The UPDRS III significantly improved from 41 [17–91] points after withdrawal of dopaminergic medication (MedOFF) to 18 [4–62] points with dopaminergic stimulation (MedON) (median [min – max]) (*p* = 0.001). After STN-DBS implantation (Table 3), patients achieved comparable motor improvement (20 [4–40]) (Fig. 1A). In the control cohort (Table 4), the preoperative motor function of patients under the MedOFF condition was assessed with 39 [23–63] points and significantly improved to 22 [8–38], in the MedON condition. In the postoperative evaluation (Table 5), STN-DBS stimulation improved motor function to 14 [1–44] (Fig. 1B). Thus, both cohorts showed comparable motor improvement by levodopa and STN-DBS.

**Table 2:** Opicapone cohort preoperative data.

ID	FOG	Falls	UPDRS I	UPDRS II	UPDRS III			MoCA	UPDRS IV				BDI-II	Hoehn-Yahr	LED
					MedOFF	MedON	Item 11 FOG		total	A	B	C			[mg]
Opi_1	yes	no	10	15	32	22	0	28	6	2	3	1	13	2.5	2393
Opi_2	yes	yes	NA	NA	36	25	2	30	NA	NA	NA	NA	2	2	1014
Opi_3	no	no	NA	NA	NA	17	NA	26	8	NA	NA	NA	11	2	1000
Opi_4	no	yes	16	15	42	18	NA	25	13	NA	NA	NA	12	4	1115
Opi_5	no	yes	1	13	91	62	0	26	7	NA	NA	NA	26	3	747
Opi_6	yes	NA	5	18	58	33	0	30	10	4	5	1	19	2.5	675
Opi_7	yes	no	12	13	41	20	0	29	9	2	7	0	9	2	1913
Opi_8	yes	yes	NA	NA	71	38	NA	24	NA	NA	NA	NA	7	3	1885
Opi_9	no	no	7	7	30	18	0	26	11	4	6	1	6	2.5	946
Opi_10	yes	no	18	15	32	18	0	24	9	0	8	1	4	2	1057
Opi_11	no	no	NA	NA	58	29	NA	26	7	3	4	0	9	2.5	2515
Opi_12	yes	no	5	5	43	18	1	30	10	4	3	3	6	2.5	1220
Opi_13	no	no	NA	NA	17	4	0	28	6	2	3	1	NA	2	1625
Opi_14	no	no	11	13	40	14	0	27	7	3	4	0	24	3	1064
Opi_15	no	no	9	14	46	15	0	28	5	0	8	0	2	2	940
Opi_16	yes	no	16	16	39	15	0	27	9	2	5	2	14	2	1370
***Median***			10	14	41	18	0	27	9	2	5	1	9	2.5	1342
***[Min-Max]***			[1–18]	[5–18]	[17–91]	[4–62]	[0–2]	[24–30]	[5–13]	[0–4]	[3–8]	[0–3]	[2–26]	[2–4]	545

FOG: freezing of gait; UPDRS: Movement Disorders Society Unified Parkinson's Disease Rating Scale; MedOFF: without dopaminergic medication; MedON: with dopaminergic medication, MoCA: Montreal Cognitive Assessment; BDI-II: Beck Depression Inventory II; LED: levodopa equivalent daily dose; NA: not available.

**Table 3:** Opicapone cohort postoperative data.

ID	Months	FOG	Falls	UPDRS III		MoCA	UPDRS IV				BDI-II	Hoehn-Yahr	LED
	post imp.			StimON	Item 11 FOG		total	A	B	C			[mg]
Opi_1	10	yes	no	33	0	NA	14	NA	NA	NA	18	2.5	1325
Opi_2	25	yes	no	32	NA	24	8	NA	NA	NA	7	2	625
Opi_3	20	no	no	17	0	25	6	3	3	0	13	2	588
Opi_4	15	no	no	21	NA	NA	NA	NA	NA	NA	8	3	575
Opi_5	36	no	NA	40	NA	27	NA	NA	NA	NA	21	3	350
Opi_6	47	no	no	38	0	30	NA	NA	NA	NA	NA	2.5	650
Opi_7	11	no	no	NA	NA	NA	NA	NA	NA	NA	6	2	1150
Opi_8	9	yes	NA	19	1	NA	6	0	4	2	NA	3	1800
Opi_9	17	yes	no	NA	NA	28	NA	NA	NA	NA	2	2	325
Opi_10	7	yes	no	22	0	NA	NA	NA	NA	NA	15	2	863
Opi_11	29	yes	no	16	0	26	6	2	2	2	NA	2.5	1420
Opi_12	8	yes	yes	34	1	29	3	0	3	0	NA	2.5	750
Opi_13	24	yes	no	14	1	NA	0	0	0	0	NA	2.5	680
Opi_14	20	no	no	4	0	24	3	0	3	0	NA	3	925
Opi_15	10	no	no	5	0	28	3	0	3	0	NA	2	840
Opi_16	21	no	no	9	0	21	12	1	7	4	NA	2	795
**Mean**	19.3		**Median**	20	0	27	6	0	3	0	11	2.5	854
**± *SD***	11.1		**[Min-Max]**	[4–40]	[0–1]	[21–30]	[0–14]	[0–3]	[0–7]	[0–4]	[[2], [3], [4], [5], [6], [7], [8], [9], [10], [11], [12], [13], [14], [15], [16], [17], [18], [19], [20], [21]]	[2,3]	396

FOG: freezing of gait; UPDRS: Movement Disorders Society Unified Parkinson's Disease Rating Scale; MedOFF: without dopaminergic medication; MedON: with dopaminergic medication, StimON: with deep brain stimulation on; MoCA: Montreal Cognitive Assessment; BDI-II: Beck Depression Inventory II; LED: levodopa equivalent daily dose; NA: not available.

**Table 4:** Control cohort preoperative data.

ID	FOG	Falls	UPDRS I	UPDRS II	UPDRS III			MoCA	UPDRS IV				BDI-II	Hoehn-Yahr	LED
					MedOFF	MedON	Item 11 FOG		total	A	B	C			[mg]
Ctrl_1	yes	yes	8	18	51	36	NA	26	0	0	0	0	17	4	1570
Ctrl_2	yes	no	4	19	40	25	NA	27	6	2	3	1	12	4	2040
Ctrl_3	no	no	1	9	23	21	NA	NA	0	0	0	0	2	2	1180
Ctrl_4	yes	NA	3	14	46	31	NA	NA	0	0	0	0	25	2.5	1270
Ctrl_5	no	no	4	13	31	20	NA	NA	0	0	0	0	16	2.5	1100
Ctrl_6	yes	yes	NA	NA	31	14	NA	29	0	0	0	0	18	3	2660
Ctrl_7	no	no	14	16	23	9	0	30	10	0	7	3	11	1	840
Ctrl_8	yes	no	4	24	57	12	0	29	14	1	10	3	3	2.5	1559
Ctrl_9	yes	NA	2	10	44	23	NA	29	3	0	3	0	8	3	1410
Ctrl_10	yes	NA	11	12	53	29	NA	29	13	3	8	2	4	3	1610
Ctrl_11	yes	no	13	19	25	8	0	30	5	0	5	0	10	2	1430
Ctrl_12	no	no	8	16	27	22	0	29	0	0	0	0	6	2	405
Ctrl_13	yes	no	14	16	52	25	0	28	6	2	3	1	9	2	1065
Ctrl_14	yes	no	7	12	63	38	0	29	14	4	6	4	14	3	1773
Ctrl_15	no	no	14	7	36	18	0	30	9	0	8	1	7	2	1118
Ctrl_16	no	no	7	15	38	19	0	28	0	0	0	0	7	2.5	500
***Median***			7	15	39	22	0	29	4	0	3	0	10	2.5	1346
***[Min-Max]***			[1–14]	[7–24]	[23–63]	[8–38]		[26–30]	[0–14]	[0–4]	[0–10]	[0–4]	[[2], [3], [4], [5], [6], [7], [8], [9], [10], [11], [12], [13], [14], [15], [16], [17], [18], [19], [20], [21], [22], [23], [24], [25]]	[[1], [2], [3], [4]]	539

FOG: freezing of gait; UPDRS: Movement Disorders Society Unified Parkinson's Disease Rating Scale; MedOFF: without dopaminergic medication; MedON: with dopaminergic medication, MoCA: Montreal Cognitive Assessment; BDI-II: Beck Depression Inventory II; LED: levodopa equivalent daily dose; NA: not available.

**Table 5:** Control cohort postoperative data.

ID	Months	FOG	Falls	UPDRS III		MoCA	UPDRS IV				BDI-II	Hoehn-Yahr	LED
	post imp.			StimON	Item 11 FOG		total	A	B	C			
Ctrl_1	9	no	yes	19	1	21	7	NA	NA	NA	26	4	130
Ctrl_2	22	yes	NA	24	1	28	NA	NA	NA	NA	6	4	2168
Ctrl_3	39	no	no	43	NA	24	0	0	0	0	1	2	400
Ctrl_4	29	yes	no	43	0	27	NA	NA	NA	NA	23	2.5	293
Ctrl_5	8	no	no	31	NA	24	0	0	0	0	6	2	400
Ctrl_6	16	no	no	3	0	25	0	0	0	0	NA	2	600
Ctrl_7	14	yes	no	14	NA	27	0	0	0	0	8	2	800
Ctrl_8	5	no	no	10	NA	NA	NA	NA	NA	NA	NA	2	930
Ctrl_9	13	yes	no	44	1	21	10	0	8	2	26	3	1073
Ctrl_10	16	no	no	1	NA	NA	8	2	5	1	NA	2	1110
Ctrl_11	20	yes	no	18	1	NA	0	0	0	0	NA	2	665
Ctrl_12	12	no	no	6	0	29	0	0	0	0	NA	2	157
Ctrl_13	8	yes	no	14	1	26	0	0	0	0	NA	2	810
Ctrl_14	19	yes	no	44	0	23	5	0	3	2	NA	3	530
Ctrl_15	28	no	no	7	0	27	6	0	6	0	NA	2	918
Ctrl_16	23	no	no	4	0	NA	0	0	0	0	NA	2.5	620
**Mean**	17.6		**Median**	14	0	25.5	0	0	0	0	8	2	725
**± *SD***	9.1		**[Min-Max]**	[1–44]	[0–1]	[21–29]	[0–10]	[0–2]	[0–8]	[0–2]	[1–26]	[2–4]	489

### 3.2 Patients treated with opicapone showed stronger motor fluctuations at shorter disease duration

**Fig. 2:**
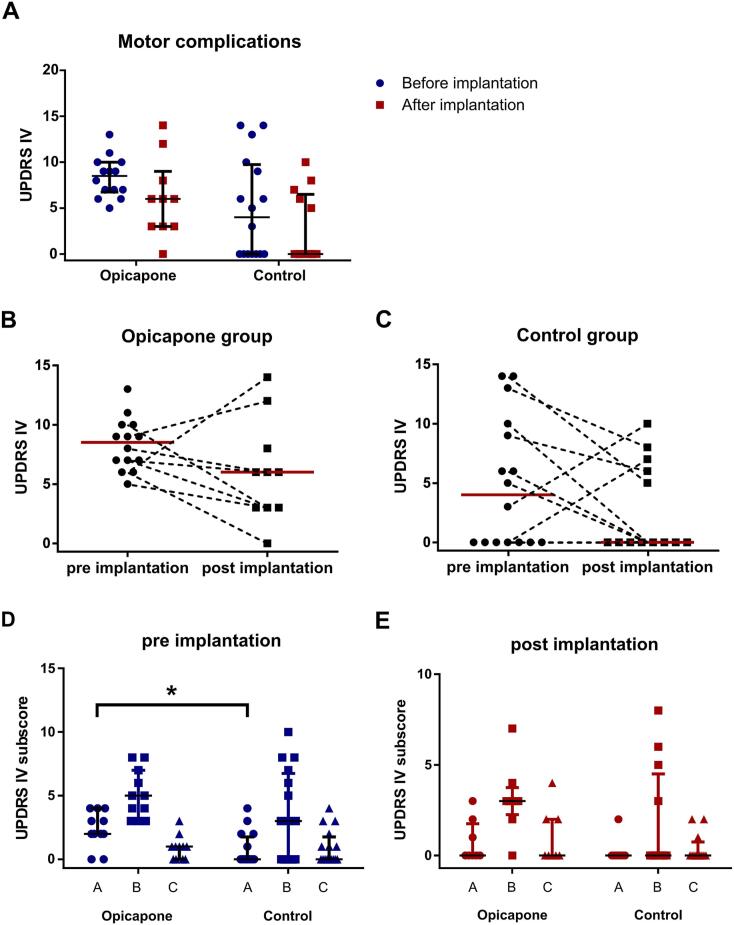
**Examination of motor complications scored by MDS-UPDRS IV. (A)** In both the opicapone and control cohorts the median MDS-UPDRS IV changed between pre- and post implantation without statistical significance. Data are displayed in scatter plots with the median and corresponding interquartile ranges. Statistical test for comparisons within the cohorts: Wilcoxon signed rank test, *p* > 0.05. **(B/C)** In the opicapone cohort, postoperative motor complications were evaluated using the MDS-UPDRS IV score in ten patients, with individual follow-up achieved for eight of these patients. In the control cohort, postoperative MDS-UPDRS IV scores were assessed in thirteen patients. Of these, five patients maintained the preoperative score of 0. Two patients experienced an increase in motor complications, while six patients showed a reduction. **(D/E)** MDS-UPDRS IV subscores (A: dyskinesias, B: OFF-state, C: painful dystonia) were compared between groups before and after STN-DBS implantation. Prior to STN-DBS implantation, patients in the opicapone cohort exhibited a higher median score for dyskinesias (UPDRS-IV-A: 2) compared to the control cohort (UPDRS-IV-A: 0), with a statistically significant difference (*p* = 0.01). Statistical test for comparisons between the cohorts: Mann-Whitney *U* test.

Motor complications of PD were assessed by the MDS-UPRDS IV scores before and after STN-DBS implantation. Preoperatively, patients in the opicapone cohort showed substantial motor complications, as indicated by MDS-UPDRS IV of 9 [5–13]. This improved after STN-DBS to 6 [0–14] without reaching statistical significance (*p* = 0.40). The patients of the control cohort showed preoperative motor complications, albeit to a lower extent 4 [0–14]. This improved with STN-DBS to a median UPDRS IV of 0 [0–10] without statistical significance (*p* = 0.33). Comparing both cohorts, patients in the opicapone cohort showed higher preoperative motor fluctuations (*p* = 0.05; Fig. 2A) although their mean disease duration (11.3 ± 3.9 years) was comparable to the control cohort (12.1 ± 6.2 years; *p* = 0.65; Table 1). With the limitation of data availability, we compared the individual development of motor fluctuations before and after STN-DBS implantation. Though the median UPDRS IV decreased in both cohorts, individual patients also showed an increase in UPDRS IV (Fig. 2B, C). Comparing the MDS-UPDRS-IV subscores, patients in the opicapone cohort were prone to a significantly higher degree of dyskinesias in the UPDRS IV-A subscore before STN-DBS implantation 2 [0–4] than patients in the control cohort 0 [0–4] (*p* = 0.01).

In conclusion, despite having a comparable disease duration, patients in the opicapone cohort exhibited dyskinesias prior to STN-DBS implantation and appeared to experience more pronounced motor complications during DBS therapy.

### 3.3 Freezing of gait

**Fig. 3:**
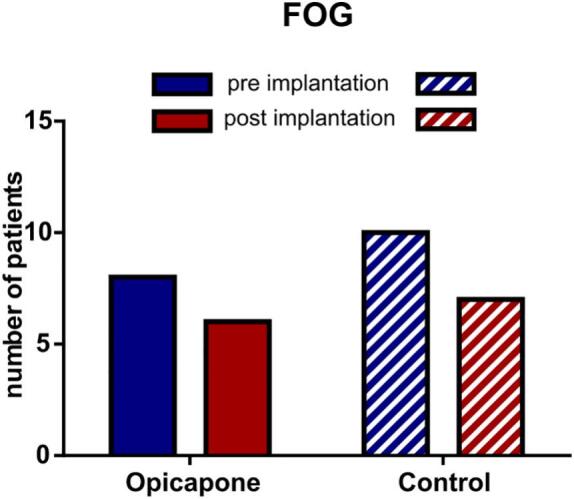
**Observation of Freezing of gait (FOG).** The patients subjected to FOG were assessed in both cohorts before and after STN-DBS implantation. In the opicapone cohort, 4/8 FOG+ were treated with opicapone before DBS implantation and 8/8 FOG+ patients were treated with opicapone postoperatively. In the control cohort, no patients were treated with opicapone. Data are displayed in bar graphs.

FOG was assessed by screening the patients' histories as well as the corresponding items in the MDS-UPDRS II and III scores. If FOG was mentioned in either the clinical documentation or the corresponding MDS-UPDRS II and III items, patients were classified as “freezers”. In the opicapone cohort, the proportion of patients with FOG remained constant, with 8 out of 16 patients affected both preoperatively and postoperatively. In the corresponding control cohort, the proportion of preoperative freezers was initially higher (10/16) and decreased to 7/16 postoperatively (Fig. 3).

### 3.4 Cognitive and psychometric properties

Cognitive function was assessed using the MoCA score. In the opicapone cohort, cognitive function did not differ significantly before 27 [24–30] and after STN-DBS implantation 27 [21−30] (*p* = 0.17). In the control cohort, preoperative cognitive function scored comparably by a median MoCA score of 27 [21–30] and increased after STN-DBS to 29 [26–30] (*p* = 0.02). Psychometric properties focusing on depressive symptoms were assessed by using the BDI-II. In the opicapone cohort, BDI-II before 9 [2–26] and after STN-DBS implantation 11 [2–21] were comparable (*p* = 0.67). In the control cohort, BDI-II improved from 10 [2–25] to 8 [1–26] without statistical significance (*p* = 0.74).

Cognitive and psychometric properties remained at a comparable level in the opicapone and control cohorts before and after STN-DBS.

### 3.5 Levodopa equivalent dose decreased after STN-DBS

**Fig. 4:**
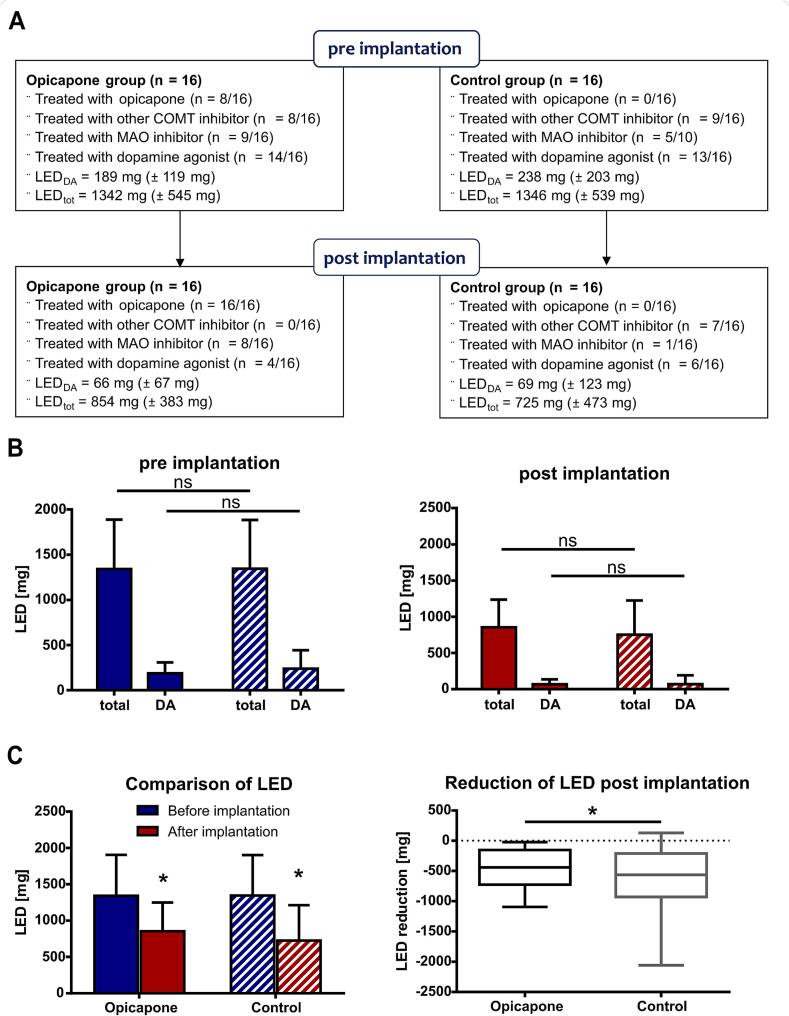
**Composition of dopaminergic medication before and after STN-DBS implantation. (A)** Flowchart comparing the composition of dopaminergic medication between the opicapone (Opi) and control (Ctrl) cohorts. **(B)** Stratification of levodopa equivalent daily dose (LED) according to total dose taken and proportion of dopamine agonists (DA). **(C)** Comparison of the LED before and after STN-DBS in the opicapone and control cohorts. The bar graphs represent the mean with error bars indicating standard deviations. The box plots represent the median extending from 25th to 75th percentiles. The whiskers represent the min and max values. Mann-Whitney *U* test was used for statistical comparisons between the cohorts and Wilcoxon signed rank test for statistical comparisons within the cohorts, p* < 0.05.

To distinguish between different types of dopaminergic medication and their respective profiles, specific drug classes administered to both cohorts were considered. All patients in the opicapone cohort were treated with any COMT inhibitor (16/16) and 9/16 patients were treated with an additional MAO inhibitor before STN-DBS implantation. Finally, after DBS implantation, all patients received opicapone and 8/16 patients were treated with a MAO inhibitor. In the control cohort, 9/16 patients were prescribed any COMT inhibitor other than opicapone before STN-DBS, which decreased to 7/16 patients using entacapone after DBS implantation. 5/16 patients in the control cohort required a MAO inhibitor preoperatively which was reduced to 1/16 after implantation (Fig. 4A). The total dopaminergic medication administered was assessed by calculating the LED. As dopamine agonists are characterised by a prolonged half-life and a different receptor profile than levodopa, they were considered separately concerning the LED. Both the opicapone and control cohorts did not differ significantly with respect to the total LED and dopamine agonist intake before and after STN-DBS implantation (Fig. 4B). Thus, a confounding effect of dopamine agonist medication was excluded.

STN-DBS allowed for a significant reduction of dopaminergic medication in both cohorts. In the opicapone cohort, the mean LED significantly decreased from 1342 ± 545 mg/d to 854 ± 396 mg/d (*p* < 0.001). The mean baseline LED administered in the control cohort was nearly identical and decreased from 1346 ± 539 mg/d preoperatively to 725 ± 489 mg/d after STN-DBS implantation (*p* = 0.001) (Fig. 4C). Comparing the reduction of dopaminergic medication in both cohorts, LED decreased by 36.4 % in the opicapone cohort, compared to 46.2 % in the control cohort representing a statistically significant difference (*p* = 0.04) (Fig. 4C).

### 3.6 DBS electrode placement was comparable in both cohorts

To ensure comparable anatomic placement of active DBS contacts between both groups, electrode placement was reconstructed. In the opicapone cohort, the active left contact was localised at lateral 12.6 mm ± 1.9 mm (mean ± SD), posterior 1.7 mm ± 2.9 mm and inferior 2.4 mm ± 2.5 mm with reference to the midcommissural point. The active right contact was localised at lateral 11.8 mm ± 2.4 mm, posterior 1.8 mm ± 2.7 mm and inferior 2.1 mm ± 2.8 mm. In the control cohort, the active left contact was localised at lateral 12.8 mm ± 2.0 mm, posterior 2.5 mm ± 2.0 mm and inferior 2.0 mm ± 2.7 mm. The active right contact was localised at lateral 12.0 mm ± 1.7 mm, posterior 2.1 mm ± 1.8 mm and inferior 2.2 mm ± 2.8 mm (Supplemental Table 2).

Given the above listed contact placement being comparable in both groups, the influence of the field of stimulation on clinical motor outcome should be without relevant difference in both groups.

## 4 Discussion

This retrospective observational study aimed to investigate the clinical role of the COMT inhibitor opicapone in the course of medication adjustments following STN-DBS implantation in a “real world clinical setting”. In this context, we investigated the clinical characteristics and outcomes of two age-matched cohorts of PD patients with and without postoperative intake of opicapone. Both cohorts were characterised by a comparable severity of motor symptoms (MDS-UPDRS III), levodopa response and dosage of dopaminergic medication (LED) before implantation. Moreover, comparable electrode placement as well as beneficial effects on functional-motor outcome assessed with the MDS-UPDRS III indicated sufficient effectiveness of STN-DBS in both cohorts.

The primary difference between the two cohorts was the presence of more pronounced motor fluctuations in the opicapone group. This was evident from a significantly higher degree of dyskinesias at baseline, as measured by the MDS-UPDRS IV-A subscore. Additionally, the opicapone group exhibited a greater degree of motor fluctuations at postoperative follow-up compared to the control group, albeit without statistical significance, likely due to the relatively small sample size. Moreover, FOG was analysed as a descriptive outcome parameter and correlate of advanced gait disorders. Patients in the opicapone cohort showed a trend of less responsiveness of freezing symptomatology following STN-DBS implantation.

Considering the cause for the difference in clinical response to STN-DBS therapy, a potential hint may be the significantly lesser extent to which dopaminergic medication could be reduced in the opicapone cohort. As the need for external dopaminergic stimulation can be seen as a proxy marker for the amount of nigrostriatal degeneration (6, 23, 24), patients in the opicapone cohort may be affected by more extensive dopaminergic denervation in spite of a slightly lower disease duration compared to controls. In this phenotype, the postoperative outcome may not only be determined by the effectiveness of STN-DBS but also by disease biology requiring a more stable dopamine replacement therapy which led to the prescription of opicapone. In the control cohort, patients also receiving COMT inhibition with entacapone, which is considered less effective in stabilising dopamine plasma levels due to its shorter half-life (12) were not excluded. Interestingly, despite this, we observed less dopaminergic dose reduction in the opicapone cohort and distinct clinical outcomes. On this basis, when treating patients showing extensive motor fluctuations including dyskinesias before STN-DBS implantation, postoperative continuation of COMT inhibition by opicapone should be considered for stabilisation of the dopaminergic stimulation.

However, the retrospective design of this analysis does not determine whether these patients were eventually benefiting from opicapone treatment as an adjunct to STN-DBS. The cohorts investigated in this study were age-matched and marked by a comparable disease duration since onset. Moreover, the anatomical placement of active DBS electrodes was comparable between both cohorts ruling out differences in electrode placement as a potential confounder. Nevertheless, the fact that in clinical practice, we select patients with motor complications for treatment with opicapone represents a selection bias in itself. Therefore, to evaluate the effectiveness of opicapone as a supplement to STN-DBS, a prospective study is needed. In such a study, a representative patient cohort should include a more sizeable case number than seen in this analysis. Moreover, as the biology of PD is considered to be the determinant for the need of stabilised dopaminergic stimulation, pathology related outcome parameters like cortical beta-wave activity (25) should be considered.

In summary, this analysis indicates that in a clinical phenotype characterised by more severe motor fluctuations and especially dyskinesias, STN-DBS needed to be supplemented by a more stable dopamine replacement therapy to address the patients' disease biology, which led to the prescription of opicapone. To analyse the clinical benefit of this therapeutic approach and to better stratify patients preoperatively into DBS and medication strategies, prospective studies are needed. This study raises hypotheses based on retrospective analyses of our single-centre cohort of STN-DBS patients and inspires novel concepts for planning enhanced therapy protocols for STN-DBS and dopamine replacement therapy.

## Funding statement

This study was funded by BIAL. The funder was not involved in the data acquisition, analysis, manuscript writing, editing, approval, or decision to publish.

FOG: freezing of gait; UPDRS: Movement Disorders Society Unified Parkinson's Disease Rating Scale; MedOFF: without dopaminergic medication; MedON: with dopaminergic medication, StimON: with deep brain stimulation on; MoCA: Montreal Cognitive Assessment; BDI-II: Beck Depression Inventory II; LED: levodopa equivalent daily dose; NA: not available.

## CRediT authorship contribution statement

**Moritz A. Loeffler:** Writing – review & editing, Writing – original draft, Visualization, Validation, Supervision, Software, Methodology, Investigation, Formal analysis, Data curation, Conceptualization. **Philipp Klocke:** Writing – review & editing, Validation. **Idil Cebi:** Writing – review & editing, Conceptualization. **Alireza Gharabaghi:** Writing – review & editing. **Daniel Weiss:** Writing – review & editing, Validation, Project administration, Funding acquisition, Conceptualization.

## Declaration of competing interest

M.A. Loeffler, P. Klocke and I. Cebi declare no financial relationships relevant to the manuscript. A. Gharabaghi received research grants from 10.13039/100001316Abbott, 10.13039/100008497Boston Scientific, 10.13039/100004374Medtronic and the German Federal Ministry of Education and Research, all not related to this work. D. Weiss reports grants and personal fees from 10.13039/100004374Medtronic, 10.13039/100001316Abbott, 10.13039/100008497Boston Scientific, all three manufacturers of DBS equipment, all not related to this work and a research grant from BIAL funding this study without participation in data analysis and manuscript preparation.

## Data Availability

The data reported in this study was acquired during routine clinical practice and may be made available in an anonymised form upon reasonable request by the authors.

## Appendix A Supplementary data

Supplemental Table 1. Localisation of active DBS contacts in relation to the midcommissural point. Image 1
