# Supplementary material for: Levodopa / opicapone as a complement to STN-DBS in clinical practice. A retrospective single-centre analysis
Source: eNeurologicalSci. 2024 Sep 28;37:100530. doi: 10.1016/j.ensci.2024.100530 (PMC11488416; doi:10.1016/j.ensci.2024.100530)
Supplement: Supplementary file 1 — Supplemental Table 1. Localisation of active DBS contacts in relation to the midcommissural point. [file mmc1.pdf]

Supplemental Table 1. Localisation of active DBS contacts in relation to the midcommissural point.

ID	Stimulation left STN	Stimulation right STN	Manufacturer	Distance [mm]	Direction	Distance [mm]	Direction	Distance [mm]	Direction	Distance [mm]	Direction	Distance [mm]	Direction	Distance [mm]	Direction
Opi_1	3-G+; 2.4mA; 60µs; 130Hz	11-G+; 1,9mA; 60µs; 130Hz	Abbott	12.6	Left	-0.8	Anterior	1.0	Inferior	10.2	Right	2.2	Posterior	1.9	Inferior
Opi_2	2-C+; 2.2mA; 60µs; 125Hz	10-C+; 2.7mA; 60µs; 125HZ	Medtronic	10.6	Left	3.5	Posterior	2.2	Inferior	11.3	Right	3.3	Posterior	2.1	Inferior
Opi_3	8-(50 %), 7- (50 %) C+;<br>2.8mA; 60 µs; 130Hz	16–(65 %) 15-(12 %) 14-(12 %) 13-(11 %) C+;<br>2.6mA; 60 µs; 130Hz	Boston	13.0	Left	0.6	Posterior	0.5	Inferior	14.4	Right	-0.7	Anterior	-0.5	Superior
Opi_4	2-C+; 3.0V; 60µs; 130Hz	11-9+; 5.5V; 60µs; 130Hz	Medtronic	13.0	Left	-1.5	Anterior	4.8	Inferior	11.2	Right	-1.3	Anterior	5.7	Inferior
Opi_5	3-C+; 2.1V; 90µs; 180Hz	11-C+; 1.1V; 60µs; 180Hz	Medtronic	13.0	Left	1.0	Posterior	2.8	Inferior	14.9	Right	0.1	Posterior	1.2	Inferior
Opi_6	2-3+; 4.3V; 90µs; 160Hz	10-11+; 4.0V; 60µs; 160Hz	Medtronic	10.9	Left	0.5	Posterior	0.4	Inferior	9.5	Right	1.4	Posterior	2.8	Inferior
Opi_7	2-3+; 1.7V; 60µs; 125 Hz	R1: 10-11+; 3.5V; 60µs; 125Hz<br>R2: 9-10+; 3.0V; 60µs; 125Hz	Medtronic	10.4	Left	6.6	Posterior	5.6	Inferior	10.1	Right	7.1	Posterior	5.2	Inferior
Opi_8	L1: 4-C+; 2.2mA; 60µs; 130Hz<br>L2: 3-C+; 1.9mA; 60µs; 130Hz	R1: 12-C+; 2.5mA; 60µs; 130Hz<br>R2: 11-C+; 2,0mA; 60µs; 130Hz	Abbott	17.5	Left	0.8	Posterior	2.6	Inferior	16.6	Right	-0.5	Anterior	-3.7	Superior
Opi_9	3C-C+; 1.4mA; 60µs; 130Hz	11B-C+; 1.9mA; 60µs; 130Hz	Abbott	11.5	Left	0.3	Posterior	2.5	Inferior	9.3	Right	1.2	Posterior	2.1	Inferior
Opi_10	2-C+; 2.1mA; 60µs; 130Hz	10-G+; 3.8mA; 60µs; 130Hz	Abbott	12.2	Left	3.1	Posterior	3.9	Inferior	9.2	Right	5.4	Posterior	5.4	Inferior
Opi_11	2A-C+; 2.7mA; 60µs; 130Hz	10A-9ABC-; C+; 1.9mA; 60µs; 130Hz	Medtronic	14.1	Left	0.2	Posterior	4.0	Inferior	12.4	Right	-0.1	Anterior	3.2	Inferior
Opi_12	3A-C+; 1.2mA; 60µs; 130Hz	11A-C+; 1.2mA; 60µs; 130Hz	Abbott	14.5	Left	-3.3	Anterior	2.6	Inferior	12.7	Right	-2.0	Anterior	2.8	Inferior
Opi_13	3-C+; 1.7mA; 60µs; 130Hz	11 C+; 1.8mA; 60µs; 130Hz	Abbott	12.0	Left	8.2	Posterior	-4.7	Superior	15.3	Right	7.3	Posterior	-4.1	Superior
Opi_14	4-C+; 4.2mA; 60µs; 130Hz	12-C+; 3.6mA; 60µs; 130Hz	Abbott	9.6	Left	0.4	Posterior	1.1	Inferior	11.3	Right	0.9	Posterior	1.1	Inferior
Opi_15	3-C+; 2.5mA; 60µs; 130Hz	11-C+; 2.6mA; 60µs; 130Hz	Abbott	14.2	Left	3.1	Posterior	3.5	Inferior	11.7	Right	1.4	Posterior	2.6	Inferior
Opi_16	2-C+; 0.9mA; 60µs; 130Hz	10-C+; 0.9mA; 60µs; 130Hz	Abbott	12.2	Left	4.0	Posterior	6.0	Inferior	8.3	Right	2.8	Posterior	5.5	Inferior
			Mean	**12.6**		**1.7**		**2.4**		**11.8**		**1.8**		**2.1**	
			± SD	**1.9**		**2.8**		**2.5**		**2.4**		**2.7**		**2.8**	
Ctrl_1	2-3+; 4.0V; 60µs; 130Hz	10-C+; 2.0V; 60µs; 130Hz	Medtronic	10.7	Left	2.8	Posterior	3.8	Inferior	9.5	Right	1.6	Posterior	2.3	Inferior
Ctrl_2	2-1+; 3.2V; 60µs; 125Hz	11-10-9+; 3.2V; 60µs; 125Hz	Medtronic	7.3	Left	4.6	Posterior	6.6	Inferior	11.4	Right	2.9	Posterior	6.3	Inferior
Ctrl_3	2-C+; 2.6V; 60µs; 130Hz	10-C+; 4.5V; 90µs; 130Hz	Medtronic	13.5	Left	1.3	Posterior	3.0	Inferior	15.0	Right	-0.5	Anterior	3.1	Inferior
Ctrl_4	2-C+; 3.0V; 120µs; 120Hz	10-C+; 3.6 V; 120µs; 120 Hz	Medtronic	13.6	Left	8.2	Posterior	-6.5	Superior	13.4	Right	7.2	Posterior	-6.6	Superior
Ctrl_5	2-C+; 5V; 60µs; 130Hz	10-C+; 4.8 V; 60µs; 130Hz	Medtronic	14.3	Left	2.1	Posterior	4.3	Inferior	12.8	Right	2.5	Posterior	3.1	Inferior
Ctrl_6	3-C+; 2.6mA; 60µs; 130Hz	11-C+; 3.5 mA; 60µs; 130Hz	Abbott	14.6	Left	2.3	Posterior	-0.5	Superior	13.8	Right	0.3	Posterior	-1.6	Superior
Ctrl_7	3C-C+; 1.7mA; 60µs; 130 Hz	12-C+; 3.7mA; 60µs; 130Hz	Abbott	11.3	Left	1.0	Posterior	1.4	Inferior	10.4	Right	-0.4	Anterior	1.4	Inferior
Ctrl_8	2-C+; 1.2mA; 60µs; 130Hz	10-G+; 1.7mA; 60µs; 130 Hz	Abbott	13.3	Left	1.9	Posterior	0.5	Inferior	13.9	Right	1.9	Posterior	1.1	Inferior
Ctrl_9	3-2+; 3.2V; 60µs; 130 Hz	10-11+; 4.7V; 60µs; 130 Hz	Medtronic	11.4	Left	4.1	Posterior	3.2	Inferior	9.5	Right	4.1	Posterior	3.8	Inferior
Ctrl_10	3-C+; 4.05mA; 60µs; 130Hz	11-C+; 4.25mA; 60µs; 130Hz	Abbott	15.5	Left	3.1	Posterior	2.4	Inferior	11.9	Right	0.3	Posterior	3.9	Inferior
Ctrl_11	3A-C+; 2.8mA; 60µs; 130Hz	11-C+; 0.5mA; 60µs; 130Hz	Abbott	11.6	Left	3.6	Posterior	2.5	Inferior	9.4	Right	2.6	Posterior	2.5	Inferior
Ctrl_12	3-C+; 3.0mA; 60µs; 130Hz	11-C+; 0.5mA; 60µs; 130Hz	Abbott	14.5	Left	2.6	Posterior	2.0	Inferior	12.6	Right	1.6	Posterior	2.0	Inferior
Ctrl_13	3-C+; 1.6mA; 60µs; 130Hz	11-C+; 1.6mA; 60µs; 130Hz	Abbott	14.1	Left	0.9	Posterior	0.7	Inferior	11.5	Right	1.2	Posterior	1.5	Inferior
Ctrl_14	2-3+; 1.7mA; 60µs; 130Hz	10-11+; 1.6mA; 60µs; 130Hz	Medtronic	11.6	Left	0.7	Posterior	4.0	Inferior	12.7	Right	2.7	Posterior	4.3	Inferior
Ctrl_15	4-C+;; 3.5mA; 60µs; 130Hz	11-C+; 2.5mA; 60µs; 130Hz	Abbott	13.3	Left	-1.4	Anterior	3.2	Inferior	11.2	Right	2.5	Posterior	3.5	Inferior
Ctrl_16	3-C+; 2.2mA; 60µs; 130Hz	10-C+; 4.7mA; 60µs; 180Hz	Abbott	14.1	Left	2.6	Posterior	1.6	Inferior	12.9	Right	3.4	Posterior	3.8	Inferior
			Mean	**12.8**		**2.5**		**2.0**		**12.0**		**2.1**		**2.1**	
			± SD	**2.0**		**2.0**		**2.7**		**1.7**		**1.8**		**2.8**	
